# Hyaluronan-enriched transfer medium in routine clinical practice: a retrospective cohort study showing no improvement in pregnancy outcomes among predominantly frozen embryo transfer cycles

**DOI:** 10.1007/s00404-026-08592-z

**Published:** 2026-10-13

**Authors:** Pelin Coştur Filiz, Serdar Filiz

**Affiliations:** 1 Department of Histology and Embryology, Faculty of Medicine, Kocaeli University, Kocaeli, Turkey; 2 Present Address: Independent Researcher, 41050 Kocaeli, Turkey; 3 Department of Histology and Embryology, Faculty of Medicine, Kocaeli University, Umuttepe Campus, 41380 İzmit, Turkey; 4 Assisted Reproduction Unit, Kocaeli University Hospital, Kocaeli, Turkey

**Keywords:** EmbryoGlue, Hyaluronan-enriched transfer medium, Frozen embryo transfer, Clinical pregnancy rate, IVF add-ons

## Abstract

**Purpose:**

To evaluate the effect of hyaluronan-enriched transfer medium (HETM; EmbryoGlue) on clinical pregnancy rates in a predominantly frozen embryo transfer (FET) cohort and to identify independent predictors of outcome.

**Methods:**

In this retrospective, nonrandomized cohort study, we analyzed 335 embryo transfer cycles at a university-based ART unit (June 2025–April 2026): HETM group (EmbryoGlue, *n* = 171) vs. control (Irvine NX medium, *n* = 164). Allocation to transfer medium was based on shared clinical decision-making. The primary outcome was clinical pregnancy rate per cycle. Multivariate logistic regression identified independent predictors.

**Results:**

FET accounted for 87.5% of cycles. Overall clinical pregnancy rate was 56.7% (190/335). Rates were comparable between HETM and control groups (56.7% vs. 56.7%; OR = 1.00, 95% CI 0.65–1.54; *p* > 0.999). No statistically significant between-group differences were observed in exploratory subgroups, including women aged ≥ 35 years, cycles with two or more previous transfer attempts, and blastocyst transfers (all *p* > 0.05). Despite a higher proportion of cycles with two or more previous transfer attempts in the HETM group (41.5% vs. 27.4%; *p* = 0.008), HETM was not an independent predictor of pregnancy (OR = 0.89, 95% CI 0.55–1.46; *p* = 0.652). FET (OR = 4.32; *p* < 0.001), blastocyst-stage transfer (OR = 2.96; *p* < 0.001), and younger age (OR = 0.92 per year; *p* = 0.001) were independently associated with clinical pregnancy.

**Conclusions:**

HETM use was not associated with higher clinical pregnancy rates in this cohort. Transfer type, embryo stage, and patient age were independently associated with clinical pregnancy. These findings do not provide evidence supporting the routine use of EmbryoGlue in unselected FET populations.

## What does this study add to the clinical work

**Table:** 

In a real-world cohort of 335 embryo transfer cycles where frozen–thawed transfer predominated, hyaluronan-enriched transfer medium (EmbryoGlue) was not associated with higher clinical pregnancy rates compared with standard medium, overall; exploratory subgroup analyses were inconclusive. Transfer type, embryo developmental stage, and patient age were associated with clinical pregnancy.	

## Introduction

In vitro fertilization (IVF) has transformed infertility treatment over the past four decades; however, successful embryo implantation remains the primary determinant of cycle outcome, with a substantial proportion of transfers failing to result in clinical pregnancy even under optimal laboratory and clinical conditions. Optimization of the embryo transfer process—including the composition of the transfer medium—therefore continues to be an active area of clinical investigation [1, 2].

Hyaluronan (hyaluronic acid, HA) is an abundant glycosaminoglycan naturally present at high concentrations in the endometrium, follicular fluid, and oviductal secretions of the female reproductive tract [3, 4]. Its principal receptor, CD44, is expressed on the surface of human embryos from the oocyte stage through blastocyst development, indicating a biologically active role in embryo–endometrium cross talk during the implantation window [5]. Experimental evidence demonstrates that hyaluronan supplementation of transfer medium promotes embryo attachment to endometrial epithelial cells in vitro and enhances fetal development following transfer in animal models [6, 7]. More recently, hyaluronan has been shown to modulate endometrial receptivity through regulation of extracellular matrix remodeling and implantation-associated signaling pathways [8]. Based on these mechanisms, EmbryoGlue (Vitrolife, Gothenburg, Sweden)—a transfer medium enriched with 0.5 mg/mL hyaluronan and reduced recombinant human albumin—was developed as a clinically applicable intervention to support embryo implantation.

Meta-analyses incorporating data from multiple randomized trials suggest a modest overall benefit of hyaluronan-enriched transfer medium (HETM) on clinical pregnancy and live birth rates [1, 2]; however, findings across individual studies remain heterogeneous. A prospective randomized study reported significantly improved implantation and clinical pregnancy rates with HETM, with the most pronounced benefit observed in women aged 35 years or older, those with previous implantation failures, and those receiving poor-quality embryos [9]. In contrast, a retrospective analysis found no significant benefit on pregnancy outcomes [10]. To our knowledge, the only double-blind randomized controlled trial conducted exclusively in frozen–thawed embryo transfer (FET) cycles demonstrated no improvement in live birth or clinical pregnancy rates with HETM compared with standard medium [11]. Collectively, these discordant findings highlight a critical evidence gap regarding HETM efficacy in predominantly FET-based clinical settings—particularly given that frozen transfer now constitutes the majority of cycles in modern ART practice. Despite this uncertainty, EmbryoGlue continues to be widely adopted as an IVF add-on, raising legitimate concerns about evidence-based practice in ART [12].

The aim of this study was to examine the association between HETM use and clinical pregnancy rates in a real-world clinical cohort in which frozen embryo transfer constituted the predominant transfer modality. We further examined pregnancy outcomes across exploratory subgroups defined by patient age, transfer type, embryo developmental stage, and number of previous transfer attempts, and identified independent predictors of clinical pregnancy using multivariate logistic regression.

## Materials and methods

### Study design and ethical approval

This retrospective cohort study was conducted at a university-based assisted reproduction unit and was reported in accordance with the STROBE (Strengthening the Reporting of Observational Studies in Epidemiology) guidelines. Data were collected from all embryo transfer cycles performed between June 2025 and April 2026. Ethical approval was obtained from the Kocaeli University Non-Interventional Clinical Research Ethics Committee (approval no. KÜ GOKAEK-2026/09/21, project no. 2026/272; date: April 22, 2026). The study was conducted in accordance with the principles of the Declaration of Helsinki. As a retrospective study involving anonymized clinical data, individual informed consent was waived.

### Study population

All women aged 18–43 years who underwent fresh (ET) or frozen–thawed (FET) embryo transfer cycles using their own oocytes during the study period were considered eligible for inclusion. Exclusion criteria were as follows: use of donor oocytes, preimplantation genetic testing (PGT) cycles, presence of a significant uterine cavity abnormality (including submucosal fibroids, endometrial polyps, or uterine septum) identified on pre-transfer assessment, and missing pregnancy outcome data. A total of 347 transfer cycles were identified; 12 were excluded due to missing outcome data, yielding a final analytic cohort of 335 cycles. The 335 analyzed cycles were contributed by 313 individual women: 292 women contributed a single cycle, 20 contributed two cycles, and one contributed three cycles. Twenty-one women (6.7%) therefore contributed more than one cycle, together accounting for 43 cycles (12.8% of the cohort).

### Ovarian stimulation and fresh embryo transfer protocol

Fresh embryo transfer cycles were performed following controlled ovarian stimulation using a flexible GnRH antagonist protocol. Fertilization was achieved by intracytoplasmic sperm injection (ICSI) in all cycles. Gonadotropin stimulation (recombinant FSH and/or human menopausal gonadotropin, 75–300 IU/day, individualized according to ovarian reserve parameters including anti-Müllerian hormone, antral follicle count, age, and body weight) was initiated on cycle days 2–3. A GnRH antagonist (cetrorelix or ganirelix, 0.25 mg/day subcutaneously) was added from stimulation day 5–6 or when a leading follicle reached ≥ 14 mm, and was continued until the day of trigger. Final oocyte maturation was triggered with recombinant hCG (250 µg subcutaneously) when the leading follicle reached ≥ 17–18 mm, or with a GnRH agonist (buserelin 0.5 mg or triptorelin 0.2 mg) in cases of elevated OHSS risk. Oocyte retrieval was performed 34–36 h after triggering. Endometrial thickness was assessed prior to transfer; a minimum thickness of 8 mm was required to proceed. Luteal phase support was initiated on the day of oocyte retrieval with vaginal progesterone (600–800 mg/day) and continued until the day of the pregnancy test. Transfer was performed on day 3 (cleavage stage) or day 5 (blastocyst stage) post-retrieval. Fresh cycles were canceled and all embryos cryopreserved when OHSS risk was elevated, endometrial thickness was insufficient, or premature progesterone elevation was detected prior to triggering.

### Frozen–thawed embryo transfer protocol

Frozen–thawed embryo transfer cycles were prepared using a standardized hormone replacement therapy protocol. Estradiol valerate was initiated on cycle days 2–3 at a dose of 2 mg/day, with stepwise increases to 4 mg/day after 5–6 days and subsequently to 6 mg/day, administered orally or vaginally over approximately 12–14 days. Endometrial development was assessed by transvaginal ultrasonography on approximately day 10–12; a minimum endometrial thickness of 8 mm with a trilaminar pattern was required to proceed. Progesterone supplementation (vaginal progesterone 600–800 mg/day or intramuscular progesterone 50 mg/day) was initiated on day P + 1, with embryo transfer performed on day P + 3 for cleavage-stage embryos and day P + 5 for blastocysts. Both estrogen and progesterone supplementation were continued until 8–10 weeks of gestation following a confirmed clinical pregnancy. All embryos had been cryopreserved by vitrification and were warmed using a standardized rapid warming protocol prior to transfer.

All embryo transfers were performed under transabdominal ultrasound guidance. Embryo morphology was assessed according to developmental stage. Cleavage-stage embryos were evaluated based on cell number, degree of fragmentation, blastomere size and symmetry, nucleation characteristics, and compaction. Blastocysts were evaluated according to the degree of expansion and the morphology of the inner cell mass and trophectoderm using the Gardner classification system. Embryo morphology was recorded for all 335 analyzed transfer cycles; stage-specific grading was not entered as a harmonized covariate in the multivariable model. A small proportion of cycles (*n* = 41, 12.2%) involved transfers on days other than day 3 or day 5, reflecting individualized clinical decisions; these were included in the analytic cohort and categorized as “Other” in embryo-stage comparisons.

### HETM application

In the HETM group, EmbryoGlue (Vitrolife, Gothenburg, Sweden) was pre-equilibrated at 37 °C and 6% CO_2_ for 4 h prior to use, in accordance with the manufacturer’s instructions. Embryos were then placed in pre-equilibrated EmbryoGlue and incubated for 1 h before transfer, and the transfer catheter was loaded with EmbryoGlue. In the control group, Irvine NX medium (Irvine Scientific, Santa Ana, CA, USA) was used as the standard embryo transfer medium throughout. HETM allocation was determined through shared clinical decision-making between the treating clinician and the patient during routine pre-transfer consultation, taking into account clinical history including prior transfer outcomes, patient preference, and clinician judgment. Neither patients nor clinicians were blinded to the transfer medium allocation.

### Outcome measures

The primary outcome was the clinical pregnancy rate per transfer cycle, defined as the presence of at least one intrauterine gestational sac confirmed by transvaginal ultrasonography at 6–7 weeks of gestation. Serum *β*-hCG was measured 10 days after transfer in day-5 cycles and 12 days after transfer in day-3 cycles; a value of ≥ 25 mIU/mL was considered positive. Secondary outcomes included clinical pregnancy rates across exploratory subgroup analyses and independent predictors of clinical pregnancy identified by logistic regression.

### Statistical analysis

Categorical variables were compared using the chi-square test or Fisher's exact test, as appropriate. Continuous variables were compared using the Mann–Whitney U test and are expressed as median with interquartile range (IQR). Odds ratios (OR) with 95% confidence intervals (CI) were calculated for binary outcomes. Multivariable logistic regression analysis was performed to examine factors independently associated with clinical pregnancy; covariates included in the model were HETM use, patient age (continuous), transfer type (FET vs. fresh ET, reference fresh ET), embryo developmental stage (day 5 vs. non-day 5, reference non-day 5), number of embryos transferred, and two or more previous transfer attempts (binary). The primary unit of analysis was the transfer cycle. Repeat transfers from the same woman were not modeled as clustered observations. To assess the potential influence of within-patient correlation, a sensitivity analysis was performed in a cohort restricted to the first transfer cycle contributed by each woman (*n* = 313), in which the unadjusted between-group comparison and the multivariable model were repeated using the same covariates as in the primary analysis. Complete case analysis was applied; 333 of the 335 cycles had complete covariate data for all model covariates and were included in the regression model (two cycles were excluded owing to a missing value for patient age). Model calibration was assessed using the Hosmer–Lemeshow test. A minimum detectable difference calculation indicated that with 171 and 164 cycles in the HETM and control groups, respectively, the study had 80% power to detect an absolute difference of 15 percentage points in clinical pregnancy rates at a two-sided significance level of 0.05, assuming a clinical pregnancy rate of 56.7% in the control group. Subgroup analyses were exploratory, were not adjusted for multiple comparisons, and were not powered to detect clinically relevant differences. Statistical significance was set at *p* < 0.05. All analyses were performed using Python (version 3.12; Python Software Foundation) with the SciPy and Statsmodels libraries.

## Results

### Patient characteristics

A total of 335 transfer cycles were included in the final analysis, comprising 171 cycles in the HETM group and 164 in the control group. The proportion of cycles contributed by women with more than one transfer was similar in the two groups (14.0% vs. 11.6%; *p* = 0.518). Baseline characteristics are summarized in Table 1. The two groups were comparable with respect to patient age, transfer type, embryo developmental stage, number of embryos transferred, and proportion of women aged 35 years or older (all *p* > 0.05). However, the HETM group had a significantly higher proportion of cycles with two or more previous transfer attempts compared with the control group (41.5% vs. 27.4%; *p* = 0.008). FET constituted 87.7% and 87.2% of cycles in the HETM and control groups, respectively. Blastocyst (day-5) transfer was performed in 57.9% and 53.0% of cycles, respectively.

**Table 1:** Baseline characteristics of the hyaluronan-enriched transfer medium (HETM) and control groups

Variable	HETM group (*n* = 171)	Control group (*n* = 164)	*p* value
Age, years, median [IQR]	30 [28–34]	31 [28–36]	0.056
Age ≥ 35 years, *n* (%)	41 (24.0)	52 (31.7)	0.143
Transfer type, *n* (%)			
Frozen–thawed (FET)	150 (87.7)	143 (87.2)	> 0.999
Fresh (ET)	21 (12.3)	21 (12.8)	
Embryo stage, *n* (%)			0.670^a^
Day 5 (blastocyst)	99 (57.9)	87 (53.0)	
Day 3 (cleavage)	52 (30.4)	56 (34.1)	
Other^b^	20 (11.7)	21 (12.8)	
Embryos transferred, median [IQR]	1 [1–2]	1 [1–2]	0.861
Previous attempts, median [IQR]	1 [1–2]	1 [1–1]	**0.008**
≥ 2 previous attempts, *n* (%)	71 (41.5)	45 (27.4)	**0.008**

Continuous variables are expressed as median [interquartile range]; categorical variables as *n* (%)

Mann–Whitney *U* test was used for continuous variables; chi-square or Fisher’s exact test for categorical variables. Significant *p* values (*p* < 0.05) are shown in bold

^a^*p* value for the overall comparison across the three embryo-stage categories (chi-square test, 2 degrees of freedom)

^b^Other includes transfers performed on days other than day 3 or day 5 (*n* = 41)

### Clinical pregnancy rates

The overall clinical pregnancy rate was 56.7% (190/335). Clinical pregnancy rates were comparable between the HETM and control groups: 56.7% (97/171) and 56.7% (93/164), respectively (OR = 1.00, 95% CI 0.65–1.54; *p* > 0.999) (Table 2). No statistically significant difference in clinical pregnancy rates was observed between the groups in the exploratory subgroup analyses, including cycles stratified by transfer type, embryo developmental stage, patient age, and number of previous attempts (all *p* > 0.05) (Table 2). These exploratory comparisons were limited by smaller subgroup sample sizes, particularly for fresh ET.

**Table 2:** Clinical pregnancy rates in the HETM and control groups: overall and by exploratory subgroups

Outcome	HETM group	Control group	OR (95% CI)	*p* value
Overall CPR, *n* (%)	97/171 (56.7)	93/164 (56.7)	1.00 (0.65–1.54)	> 0.999
CPR—FET cycles, *n* (%)	90/150 (60.0)	88/143 (61.5)	0.94 (0.59–1.50)	0.812
CPR—ET cycles, *n* (%)^a^	7/21 (33.3)	5/21 (23.8)	1.60 (0.41–6.19)	0.734
CPR—day 5 transfers, *n* (%)	67/99 (67.7)	63/87 (72.4)	0.80 (0.42–1.50)	0.524
CPR—day 3 transfers, *n* (%)	17/52 (32.7)	18/56 (32.1)	1.03 (0.46–2.30)	> 0.999
CPR—Age < 35 years, *n* (%)	81/129 (62.8)	72/111 (64.9)	0.91 (0.54–1.55)	0.788
CPR—age ≥ 35 years, *n* (%)	16/41 (39.0)	20/52 (38.5)	1.02 (0.44–2.37)	> 0.999
CPR—1st attempt, *n* (%)	51/90 (56.7)	56/103 (54.4)	1.10 (0.62–1.94)	0.773
CPR—≥ 2 attempts, *n* (%)	39/71 (54.9)	25/45 (55.6)	0.97 (0.46–2.07)	> 0.999

*CPR* clinical pregnancy rate, *OR* odds ratio, *CI* confidence interval, *FET* frozen–thawed embryo transfer, *ET* fresh embryo transfer

^a^Results for fresh embryo transfer (ET) cycles should be interpreted with caution owing to the small sample size (*n* = 42). Age subgroup denominators do not sum to the group totals because patient age was missing in two cycles (one in each group). All subgroup comparisons are exploratory and were not adjusted for multiple testing

In the FET subgroup (*n* = 293, 87.5% of all cycles), clinical pregnancy rates were 60.0% (90/150) in the HETM group and 61.5% (88/143) in the control group (OR = 0.94, 95% CI 0.59–1.50; *p* = 0.812). Among blastocyst transfers, rates were 67.7% (67/99) and 72.4% (63/87) in the HETM and control groups, respectively (OR = 0.80, 95% CI 0.42–1.50; *p* = 0.524). In women aged 35 years or older, clinical pregnancy rates were 39.0% (16/41) versus 38.5% (20/52) (OR = 1.02, 95% CI 0.44–2.37; *p* > 0.999). Among cycles with two or more previous transfer attempts, rates were 54.9% (39/71) and 55.6% (25/45) in the HETM and control groups, respectively (OR = 0.97, 95% CI 0.46–2.07; *p* > 0.999).

### Multivariate logistic regression

Multivariate logistic regression analysis (*n* = 333) identified frozen–thawed transfer (FET vs. ET: OR = 4.32, 95% CI 2.00–9.31; *p* < 0.001), blastocyst-stage transfer (day 5 vs. other: OR = 2.96, 95% CI 1.82–4.82; *p* < 0.001), and younger patient age (per year: OR = 0.92, 95% CI 0.88–0.96; *p* = 0.001) as independent predictors of clinical pregnancy. HETM use was not an independent predictor of clinical pregnancy (OR = 0.89, 95% CI 0.55–1.46; *p* = 0.652). Number of embryos transferred and history of previous transfer attempts were also not independently associated with outcome. The model demonstrated good discrimination (AUC = 0.734) and borderline acceptable calibration (Hosmer–Lemeshow test, *p* = 0.053). Full results of the multivariate analysis are presented in Table 3. In the sensitivity analysis restricted to the first transfer cycle contributed by each woman (*n* = 313), clinical pregnancy rates were 54.3% (88/162) in the HETM group and 56.3% (85/151) in the control group (OR = 0.92, 95% CI 0.59–1.44; *p* = 0.734), and HETM was again not independently associated with clinical pregnancy (model n = 311; OR = 0.83, 95% CI 0.50–1.37; *p* = 0.466).

**Table 3:** Multivariate logistic regression analysis: independent predictors of clinical pregnancy

Variable	OR	95% CI	*p* value
HETM vs. control	0.89	0.55–1.46	0.652
Patient age (per year)	0.92	0.88–0.96	0.001
Transfer type (FET vs. ET)	4.32	2.00–9.31	< 0.001
Embryo stage (day 5 vs. other)	2.96	1.82–4.82	< 0.001
Number of embryos transferred	0.92	0.69–1.24	0.594
≥ 2 previous transfer attempts	0.84	0.50–1.41	0.512

Model *n* = 333; AUC = 0.734; Nagelkerke *R*^2^ = 0.131; Hosmer–Lemeshow *p* = 0.053

*OR* odds ratio, *CI* confidence interval

Fresh embryo transfer (ET) and non-day 5 embryo stage were used as reference categories for the respective binary variables. Age was modeled as a continuous variable; OR represents the change in odds per 1-year increase in age

## Discussion

In this real-world retrospective cohort study of 335 embryo transfer cycles, the use of HETM was not associated with improved clinical pregnancy rates compared with standard transfer medium, in the overall cohort; exploratory subgroup comparisons also did not show statistically significant differences, including women aged 35 years or older, cycles with two or more previous transfer attempts, and blastocyst transfers. Multivariate logistic regression found that HETM use was not independently associated with clinical pregnancy (OR = 0.89; *p* = 0.652), whereas frozen–thawed transfer type, blastocyst-stage embryo, and younger patient age were independently associated with clinical pregnancy. These findings contribute real-world evidence to the ongoing debate regarding the clinical utility of EmbryoGlue, particularly in cohorts where frozen embryo transfer constitutes the predominant modality.

Our results contrast with several earlier studies reporting a benefit of HETM, although the randomized evidence is not uniform. Nakagawa et al. demonstrated improved outcomes with HETM specifically in patients with repeated implantation failure [13], whereas Fancsovits et al., in a prospective randomized trial of 581 cycles, found no beneficial effect of hyaluronan enrichment on clinical pregnancy, implantation or delivery rates [14]. Adeniyi et al. found a significant benefit of hyaluronate-containing medium on clinical pregnancy and live birth outcomes in a large single-center cohort [15], and Korošec et al., comparing single fresh elective and frozen–thawed blastocyst transfers, found overall pregnancy rates to be similar, with a significant advantage for hyaluronan confined to a selected subgroup of fresh elective transfers: women with at least two blastocysts developed to day 5 and a previous implantation failure (55% versus 10%; *p* = 0.012) [16]. Notably, the most frequently cited positive study—Urman et al.—reported an overall increase in implantation and clinical pregnancy rates, with the largest effects in women aged 35 or older, those with prior implantation failures, and those with poor-quality embryos [9]. In the present study, no statistically significant differences were observed within these exploratory subgroups. However, the limited subgroup sample sizes do not exclude a clinically relevant effect in selected populations.

Evidence regarding the use of HETM in frozen embryo transfer cycles remains mixed. Yan et al. [17] reported a significantly higher clinical pregnancy rate with HETM in patients with recurrent implantation failure undergoing FET, although the difference in live birth rates did not reach statistical significance. Conversely, Horiuchi et al. [18] found no statistically significant difference in clinical pregnancy rates following low-grade frozen embryo transfers, while Greco et al. [19] reported no statistically significant improvement in clinical pregnancy or live birth rates following frozen single euploid blastocyst transfers after PGT-A. At a broader level, a systematic review and meta-analysis by Heymann et al. [20] reported increased clinical pregnancy and live birth rates with HA-enriched transfer medium in autologous oocyte cycles, with estimated live birth rates of 32% and 39% in the control and HA-enriched groups, respectively (RR 1.22, 95% CI 1.11–1.34). No statistically significant benefit was demonstrated in donor oocyte cycles. Although these findings contrast with those of the present cohort, the meta-analysis included both fresh and frozen embryo transfers. More recently, Toyoda et al. [21] reported a single-center retrospective historical-cohort study of 726 FET cycles in which clinical pregnancy rates were higher with HETM than with Hyaluronan-free transfer medium (37.7% vs. 27.2%; *p* = 0.002). This association was particularly pronounced in cycles with recurrent implantation failure, defined as failure to achieve pregnancy after at least two previous embryo transfers (adjusted OR 2.4, 95% CI 1.4–3.9). Although the historical-cohort design and differences in baseline clinical pregnancy rates limit direct comparison with our population, these findings further support the possibility that selected patient groups may benefit from HETM, which our exploratory analyses cannot exclude. Taken together, the available evidence suggests that findings may differ across study populations and transfer settings, and the present results should be interpreted within this broader context.

The mechanistic rationale for HETM use was initially grounded in the observation that HA could effectively substitute for albumin as the principal macromolecule in embryo transfer media, thereby more closely mimicking the viscous environment of the uterine cavity [22, 23]. In fresh transfer cycles, where the endometrial environment may be suboptimal due to supraphysiological estrogen levels following ovarian stimulation, the supplementary effects of hyaluronan on embryo–endometrium adhesion may be more clinically meaningful. In contrast, the standardized hormone replacement protocols used in FET cycles in the present study yielded a high baseline clinical pregnancy rate of 61.5% in the control group FET cycles—a level at which the incremental benefit of any adjunct may be more difficult to demonstrate statistically. This interpretation is further supported by the observation that frozen–thawed transfer was associated with a fourfold higher odds of clinical pregnancy compared with fresh transfer in the present cohort (OR = 4.32), although selection and other clinical differences between fresh and frozen transfer cycles may also contribute to this association.

A notable and clinically relevant finding of the present study was the significant difference between the two groups in the proportion of cycles with two or more previous transfer attempts: 41.5% in the HETM group compared with 27.4% in the control group (*p* = 0.008). This imbalance reflects an intuitive clinical practice—the escalation of add-on interventions in patients who have not responded to standard treatment—that is well recognized in the ART literature [24, 25]. Among cycles with two or more previous transfer attempts, no statistically significant between-group difference in clinical pregnancy was observed (54.9% vs. 55.6%; *p* > 0.999). The interpretive consequences of this imbalance run in two directions. HETM was applied preferentially to a prognostically less favorable population, which nonetheless achieved clinical pregnancy rates comparable to those of the control group. Confounding by indication of this kind would be expected to bias the observed association of HETM downward, so that a true benefit would be underestimated and an apparent harm exaggerated; the present null estimate should therefore not be interpreted as evidence that HETM is harmful. The HETM group was also enriched for the population in which a benefit of hyaluronan has most often been claimed, and no advantage was apparent in this group, an observation consistent with our overall finding. Conversely, this is also the group in which our data are least informative, since only 116 cycles involved two or more previous transfer attempts and the study was not powered to detect effects within subgroups. For patients with recurrent implantation failure specifically, systematic reviews and network meta-analyses have identified several evidence-based interventions; HETM does not consistently rank among those with demonstrable efficacy in this population [26]. Fu et al. nevertheless reported improved implantation and clinical pregnancy rates with hyaluronan-enriched medium among women undergoing a third or subsequent FET attempt, while recommending caution in first and second attempts [27]. The present exploratory comparison cannot exclude such an effect in selected patients, and our findings should not be read as evidence against a benefit of hyaluronan in women with recurrent implantation failure.

Several limitations of this study warrant acknowledgment. The retrospective, nonrandomized design and the absence of blinding are inherent constraints that introduce the risk of selection bias, despite adjustment for key confounders in the logistic regression model. The potential confounding effect of differential HETM selection in patients with previous transfer attempts was addressed by including the number of previous transfer attempts as a covariate in the multivariate model; nonetheless, residual confounding cannot be excluded. Infertility diagnosis was not systematically recorded and could not be included as a covariate in the regression model. The single-center setting limits generalizability. Embryo morphology was recorded for all analyzed transfer cycles, but stage-specific grading  was neither harmonized nor included in the multivariable model; residual confounding related to embryo quality therefore remains possible. Repeated transfers from the same woman were not modeled as clustered observations in the primary cycle-level analysis. A sensitivity analysis restricted to the first cycle contributed by each woman, in which within-patient correlation cannot arise, yielded results consistent with the primary analysis, although this analysis included fewer cycles and was correspondingly less precise. Clinical pregnancy was used as the primary endpoint. Our unit is a regional referral center, and most women return to their home province for antenatal care once a pregnancy is confirmed. Miscarriage, ongoing pregnancy and live birth were therefore not recorded in a structured, retrievable form and could not be systematically ascertained across the cohort; where information on later outcomes existed, it was incidental rather than systematically collected. Outcomes beyond clinical pregnancy were consequently not analyzed. Additionally, the incubation protocol used in the present study (4 h of medium pre-equilibration followed by 1 h of embryo incubation in EmbryoGlue) differs from shorter incubation periods reported in some published studies (e.g., 30 min); such differences in protocol may influence outcomes and limit comparability across studies. Finally, the study was powered to detect an absolute difference of 15 percentage points; smaller but potentially clinically meaningful differences cannot be excluded. The fresh ET subgroup was small, and all subgroup analyses should be regarded as exploratory rather than evidence of equivalence between media. The absence of a statistically significant difference should not be interpreted as evidence that no clinically relevant effect exists.

In conclusion, HETM use was not associated with improved clinical pregnancy rates in this real-world cohort of predominantly frozen embryo transfer cycles, overall; no statistically significant differences were observed in exploratory subgroups. Transfer type, embryo developmental stage, and patient age were independently associated with clinical pregnancy in the adjusted analysis. These findings do not provide evidence supporting the routine use of EmbryoGlue in unselected FET populations and highlight the importance of evidence-based evaluation of ART add-ons prior to widespread clinical adoption. Prospective randomized trials with live birth as the primary endpoint remain warranted to definitively characterize any potential benefit of HETM in specific patient subgroups.

## Data Availability

The datasets generated and analyzed during the current study are not publicly available due to patient privacy considerations but are available from the corresponding author on reasonable request.
